# Autophagy Blockage Up-Regulates HLA-Class-I Molecule Expression in Lung Cancer and Enhances Anti-PD-L1 Immunotherapy Efficacy

**DOI:** 10.3390/cancers16193272

**Published:** 2024-09-26

**Authors:** Erasmia Xanthopoulou, Ioannis Lamprou, Achilleas G. Mitrakas, Georgios D. Michos, Christos E. Zois, Alexandra Giatromanolaki, Adrian L. Harris, Michael I. Koukourakis

**Affiliations:** 1 Department of Radiotherapy/Oncology, University Hospital of Alexandroupolis, Democritus University of Thrace, 68100 Alexandroupolis, Greece; erxantho@med.duth.gr (E.X.); ilamprou@med.duth.gr (I.L.); amitrak@med.duth.gr (A.G.M.); geormich30@med.duth.gr (G.D.M.); christos.zois@oncology.ox.ac.uk (C.E.Z.); 2 Department of Oncology, University of Oxford, Old Road Campus Research Building, Roosevelt Drive, Oxford OX3 7DQ, UK; adrian.harris@oncology.ox.ac.uk; 3 Department of Pathology, University Hospital of Alexandroupolis, Democritus University of Thrace, 68100 Alexandroupolis, Greece; agiatrom@med.duth.gr

**Keywords:** lung cancer, autophagy, HLA-class-I, chloroquine, LC3A, immunotherapy

## Abstract

**Simple Summary:**

The restoration of HLA-class-I expression in lung cancer cells may reverse immune evasion, enhance cytotoxic T-cell activity, and improve immune checkpoint inhibitors’ therapeutic efficacy. In the current study, we provide experimental evidence that autophagy blockage either at the late stage (chloroquine and bafilomycin) or early stage of the autophagic process (ULK1 inhibitors and MAP1LC3A silencing) can up-regulate the expression of HLA-class-I molecules in the A549 and H1299 NSCLC cell lines and their CD133+ stem cells. This upregulation enhances the cytotoxic activity of activated CD8+ T-cells, in particular after pre-incubation with anti-PD-L1 monoclonal antibodies. The restoration of HLA-class-I-mediated antigen presentation with autophagy blockers in lung cancer is a promising avenue that urgently requires further exploration in clinical trials.

**Abstract:**

Background/Objectives: Immune checkpoint inhibitors have an established role in non-small cell lung cancer (NSCLC) therapy. The loss of HLA-class-I expression allows cancer cell evasion from immune surveillance, disease progression, and failure of immunotherapy. The restoration of HLA-class-I expression may prove to be a game-changer in current immunotherapy strategies. Autophagic activity has been recently postulated to repress HLA-class-I expression in cancer cells. Methods: NSCLC cell lines (A549 and H1299) underwent late-stage (chloroquine and bafilomycin) and early-stage autophagy blockage (ULK1 inhibitors and MAP1LC3A silencing). The HLA-class-I expression was assessed with flow cytometry, a Western blot, and RT-PCR. NSCLC tissues were examined for MAP1LC3A and HLA-class-I expression using double immunohistochemistry. CD8+ T-cell cytotoxicity was examined in cancer cells pre-incubated with chloroquine and anti-PD-L1 monoclonal antibodies (Moabs); Results: A striking increase in HLA-class-I expression following incubation with chloroquine, bafilomycin, and IFNγ was noted in A549 and H1299 cancer cells, respectively. This effect was further confirmed in CD133+ cancer stem cells. HLA-class-I, β2-microglobulin, and TAP1 mRNA levels remained stable. Prolonged exposure to chloroquine further enhanced HLA-class-I expression. Similar results were noted following exposure to a ULK1 and a PIKfyve inhibitor. Permanent silencing of the MAP1LC3A gene resulted in enhanced HLA-class-I expression. In immunohistochemistry experiments, double LC3A+/HLA-class-I expression was seldom. Pre-incubation of H1299 cancer cells with chloroquine and anti-PD-L1 MoAbs increased the mean % of apoptotic/necrotic cells from 2.5% to 18.4%; Conclusions: Autophagy blockers acting either at late or early stages of the autophagic process may restore HLA-class-I-mediated antigen presentation, eventually leading to enhanced immunotherapy efficacy.

## 1. Introduction

Non-small cell lung cancer (NSCLC) is one of the most common and lethal tumors worldwide. Due to the high rate of locally advanced and metastatic disease at diagnosis, or even the frequent presence of adverse medical conditions, the vast majority of cases are inoperable and are treated with chemotherapy or chemo-radiotherapy (CRT) [1]. The addition of post-CRT immunotherapy and the combination of chemotherapy with anti-PD-1/PD-L1 monoclonal antibodies have significantly improved the survival rates of this devastating disease [2,3]. The encouraging results of trials with immune checkpoint inhibitors (ICIs) in NSCLC confirm the immunological background of the disease and suggest that antitumor immunity has the potential to eradicate this tumor under certain conditions. Unfortunately, about 60% of tumors will not respond to ICIs, and even for responders, the median time to relapse barely exceeds two years [4].

Multiple causes have been suggested to explain de novo resistance and development of refractory disease during immunotherapy, including microenvironmental conditions leading to T-cell exhaustion, compromised systemic immunological state, and also defective neoantigen presentation by the human major histocompatibility complex (MHC/HLA) [5]. A study by Wang et al. suggested that the development of resistance to anti-PD-1 immunotherapy in mice is directly related to loss of MHC-class-I antigen expression, while tumor irradiation that induces an IFN-type-I response restores their expression and reverses resistance [6]. In a previous study, we showed that the complete loss of HLA-class-I expression occurs in 41.5% of human NSCLCs, while an extensive loss is also evident in an additional 23.4% of cases [7]. It is therefore postulated that primary HLA under-expression or the subsequent loss of expression during immunotherapy may be an important component of failure to respond to ICIs. The constitutive loss of expression occurs due to multiple mechanisms, such as mutations of essential genes like β2-microglobulin [8], which may explain HLA-class-I repression. However, suppression of HLA-class-I is more frequently a result of an orchestrated phenomenon involving the suppression of HLA, β2-microglobulin, and antigen transporter genes. This process strongly depends on microenvironmental conditions and exposure to cytokines and is reversible [6,7,9].

Finding novel therapeutic approaches to restore HLA-class-I lost expression in NSCLC could prove important in augmenting the efficacy of ICIs or even re-establishing their effectiveness once tumors have developed resistance. Autophagy is a major cellular process activated in NSCLC, and as previously reported by our group, specific patterns of MAP1LC3A (LC3A for simplicity) expression are related to poor prognosis, while the suppression of LC3A-mediated autophagy sensitizes lung cancer cells to radiotherapy and chemotherapy [10,11]. Recent experimental evidence suggests that autophagy promotes the repression of HLA-class-I and evasion from immune surveillance [12].

In the current study, we provide experimental evidence that autophagy blockage with various drugs and molecular targeting strategies can up-regulate the expression of HLA-class-I molecules in NSCLC, thereby enhancing the cytotoxic activity of activated T-cells.

## 2. Materials and Methods

### 2.1. Cell Lines and Cell Culture Conditions 

Cell lines A549 (human alveolar basal epithelial adenocarcinoma cells) and H1299 (human non-small cell lung carcinoma cells) were purchased from ATCC (ATCC CCL-185 and ATCC CRL-5803, respectively). Details are available at http://www.atcc.org/Products/Cells_and_Microorganisms/Testing_and_Characterization/STR_Profiling_Analysis.aspx (accessed on 23 September 2024). All cell lines were cultured under aseptic conditions using DMEM Low Glucose (Dulbecco’s Modifed Eagle Medium; Biosera, Cholet, France], supplemented with 10% (*v*/*v*) 10% fetal bovine serum (PAN-Biotech, DE, Aidenbach, Germany) and 1% (*v*/*v*) 1% penicillin/streptomycin [Cat no: 15140-122; Gibco, Life Technologies, Waltham, MA, USA]. Cell cultures were maintained under standard conditions at 37 °C with 5% CO_2_ in a humidified incubator. Το ensure the consistency and reproducibility of results in experiments involving cell lines, at least 3 biological replicates were treated identically. Quality control measures such as mycoplasma testing, calibrated equipment, and blinded analysis were applied throughout each experimental process described in the current study. These steps prevent biased results during data analysis and ensure reliable and consistent experimental results.

### 2.2. Treatment Conditions with Autophagy-Targeting Agents

For the evaluation of the expression pattern of HLA-class-I protein, lung cancer cell lines were treated with 10 μΜ chloroquine [Sigma-Aldrich, Burlington, MA, USA] diluted in DMEM Low Glucose [Biosera, Cholet, France], 100 nM bafilomycin A1 [Cayman Chemical Company, Ann Arbor, MI, USA], 30 nM NVP-BEZ235/Dacrtolisib [Santa Cruz, Dallas, TX, USA], and 25 ng/mL IFN-γ [Recombinant Human IFN-γ; PeproTech, Cranbury, NJ, USA]. Moreover, the A549 and the H1299 cell lines were exposed to other autophagy inhibitors as follows: i. 2.5 μΜ of SBI-0206965 ULK1 inhibitor [MedKoo Biosciences, Durham, NC, USA]; ii. 5 μΜ of YM-201636 (PIKfyve inhibitor) [APExBIO, Hsinchu, Taiwan]; and iii. 300 nM of PIK5-12D (PROTAC PIKfyve degrader) [MedChemExpress, Monmouth Junctio, NJ, USA] for 72 h.

Chloroquine is an inhibitor of autophagy flux that impairs autophagosome–lysosome fusion accompanied with severe disorganization of the Golgi and endo-lysosomal systems [13]. Bafilomycin A1 blocks autophagy flux by inhibiting lysosomal degradation capacity through the reduction in their acidity, and it can also impair autophagosome/lysosome fusion [14]. NVP-BEZ235/Dactolisib is a dual PI3K/mTOR inhibitor, indirectly inducing autophagy [15]. The compound SBI-0206965 is a highly selective inhibitor of the autophagy-promoting serine/threonine kinase ULK-1 (Unc-51-like kinase 1) [16]. This inhibitor suppresses ULK-1-mediated phosphorylation events in cells and prevents ULK-1-dependent cell survival by suppressing autophagy [17,18]. Additionally, it has been demonstrated that this compound induces apoptosis in non-small cell lung cancer (NSCLC) through mechanisms independent of autophagy, which was partly mediated by the destabilization of Bcl2/Bclxl, and in clear cell renal cell carcinoma by increasing the levels of reactive oxygen species (ROS). YM201636 is a potent and selective PIKfyve inhibitor. PIK5-12d is a proteolysis targeting chimera (PROTAC) specific for PIKfyve, which is a lipid kinase, responsible for the main pool of PI5P and the only known enzyme for the production of PI(3,5)P2. PIKfyve’s main role is the maturation of early into late endosomes and lysosomes, retrograde vesicle transport to the trans Golgi network, and is implicated in various cellular mechanisms such as autophagy and exocytosis [19,20,21].

### 2.3. Development of Stably Transfected Lung Cancer Cell Lines

The human lung carcinoma H1299 cell line was stably transfected with a non-coding (shNC) plasmid vector or a shLC3A plasmid vector (Shanghai GenePharma, Shanghai, China) containing the geneticin resistance gene, along with a gene responsible for expressing the fluorescent mCherry protein, as previously described by our lab [11]. Furthermore, the transfected cells (shNC or shLC3A) were sorted by flow cytometry to increase the percentage of positive cells that gradually accumulated into the culture. Microscopy images were acquired with the Cell IQ imaging system (×10 magnification) in order to evaluate any morphological changes in the aforementioned lung cancer cell lines post-stable transfection.

### 2.4. Western Blot

Each lung cancer cell line was seeded in a T-75 flask at 50% confluency the day prior to treatment with chloroquine, bafylomycin A, NVP-BEZ235, or ionizing radiation. Cell lysates were collected using RIPA lysis buffer (cat# SLCD5849, Sigma-Aldrich, USA) with protease/phosphatase inhibitors (cat# 20-201/cat# 524629, EMD Millipore Corporation, Burlington, MA, USA) for 15 min on ice under frequent agitation, followed by scraping and manual syringe/needle (20-gauge) homogenization for at least 5–10 times until a homogeneous lysate was achieved. Protein concentration was determined using the DCTM protein assay kit (BioRad, Hercules, CA, USA). Protein samples (25 μg) were resolved by discontinuous SDS-PAGE (10–12%, depending on the studied protein) and transferred onto 0.45 um pore size PVDF membranes (Merck-Millipore, Burlington, MA, USA) at 120 mA for 90 min at 4 °C. Subsequently, membranes were blocked with 5% non-fat dried milk in TBS-T buffer for 1 h at RT, and incubated O/N at 4 °C with the appropriate primary antibody. HLA-class-I (ABC) molecule expression was detected using the mouse monoclonal primary antibody (1:1000, Abcam, Cambridge, UK) and the PD-L1 protein was detected with the rabbit monoclonal anti-PD-L1 [clone CAL10] primary antibody (1:1000, BiocareMedical, Concord, CA, USA). Next, membranes were incubated with a goat anti-mouse HRP-conjugated (Biorad, Hercules, CA, USA, Watford, UK) or a goat anti-rabbit HRP-conjugated secondary antibody (BioRad, UK) at a dilution of 1:1000 for 1 h at RT, followed by detection of the chemiluminescent signal using ECL substrates (Thermo-Fisher Scientific Inc., Waltham, MA, USA). Each membrane was stripped and re-hybridized with the mouse monoclonal antibody against beta-actin to confirm equal loading of samples (1:5000, NB 600–501, Novus Biologicals, Centennial, CO, USA). Immunoblot images were developed using the Chemidoc^®^ MP imaging system (BioRad, USA) and band intensity was analyzed with the ImageLab software v6.1 (BioRad, USA).

### 2.5. RNA Isolation, cDNA Synthesis, and qPCR

The total RNA of untreated and treated cells was isolated using Nucleospin^®^ RNA Plus Kit (Macherey-Nagel, Duren, Germany). RNA purity (260/280 nm and 260/230 nm ratios) and concentration were evaluated and performed using the NanoDrop 2000c (Thermo Fisher Scientific). Total RNA (500 ng) was used to synthesize cDNA utilizing the PrimeScriptTM RT Reagent Kit (RR037A, Takara, Osaka, Japan) according to the manufacturer’s protocol. The reaction steps included incubation at 37 °C for 15 min and 85 °C for 5 s. Both steps were performed in a thermal block cycler with a heated lid (Mastercycler^®^ pro, Eppendorf, Hamburg, Germany). qPCR was performed using the KAPA SYBR^®^ FAST qPCR Kit optimized for Light Cycler^®^ 480 (KK4611; Kapa Biosystems, Wilmington, MA, USA) on a LightCycler^®^ 480 Instrument II (Roche, Rotkreuz, Switzerland).

qPCR primer sets were designed using the Roche primer design tool as follows: human HLA forward 5′-CCT-ACG-ACG-GCA-AGG-ATT-AC-3′ and reverse 5′-GC-CAG-GTC-AGT-GTG-ATC-TC-3′; human TAP1 forward 5′-GGA-CCA-CTA-GTA-TTT-CAG-GTA-TGC-3′ and reverse 5′-GAG-CAG-TAC-CTC-CAC-AGC-C-3′; and β2m forward 5′-TTC-TGG-CCT-GGA-GGC-TAT-C-3′ and reverse 5′-TCA-GGA-AAT-TTG-ACT-TTC-CAT-TC-3′ [22,23]. For normalization purposes, the housekeeping gene HPRT1 was included in our study, with the following primer set used for its amplification: forward 5′-TGA-CCT-TGA-TTT-ATT-TTG-CAT-ACC-3′ and reverse 5′-CGA-GCA-AGA-CGT-TCA-GTC-CT-3′.

Cycling conditions for qPCR consisted of four steps, pre-incubation at 95 °C for 3 min; amplification for 40 cycles at 95 °C for 10 s, 58 °C for 20 s, and 72 °C for 1 s; melting curve steps at 95 °C for 5 s and 65 °C for 1 min; and cooling at 40 °C for 10 s. Quantitative analysis was performed using LightCycler software (LightCycler^®^ 480 SW 1.5.0 SP4 v1.5.0.39, Roche, Rotkreuz, Switzerland), where cycle thresholds (Cts) were determined for the genes of interest and then normalized to HPRT1 using the ΔΔCt method. The experiments were performed in triplicate and repeated three times.

### 2.6. Flow Cytometry

Flow cytometry was performed to assess the expression of HLA-class-I protein in lung cancer cell lines. Following exposure to the aforementioned agents and in the described conditions, cells were trypsinized, collected, centrifuged, and washed with PBS 1x [Biosera, Cholet, France, Lot no: 017BS454]. Thereafter, incubation with an HLA-class-I ABC [ab33257; PE Anti- HLA-Class-I antibody; Abcam, UK] antibody and a CD133 antibody [Mouse Human APC; 293C3; 567909 APC- Mouse IgG2b, Becton Dickinson (BD), New Jersey, USA] for 30 min was performed. Moreover, double staining with an HLA-class-I ABC [ab33257; PE Anti- HLA-Class-I antibody; Abcam, UK] antibody and a CD133 antibody [Mouse Human APC; 293C3; 567909 APC- Mouse IgG2b Becton, Dickinson (BD), New Jersey, USA] for 30 min was performed. The analysis of the samples was conducted using the BD FACSAria™ III system and FlowJo V10 software was used for the analysis of all the data. The gating strategy was based on the unstained control condition of each cell line and all experiments were performed three times. The stop recording point was set at 30,000 events.

### 2.7. CD8+ T-Cell Sorting and Lymphotoxicity Assay

For the current experimental procedure, peripheral blood mononuclear cells (PBMCs) were extracted from blood samples of a healthy donor, utilizing Lymphosep [Lymphocyte Separation Media; density 1077 g/mL; Biosera, Cholet, France]. Cells were washed twice with PBS 1x [Biosera, Cholet, France] and then stained with the CD3-BV421 antibody [Brilliant Violet 421TM anti-human CD3 Antibody; Clone: OKT3; BioLegend, San Diego, CA, USA] and the CD8- PerCP-Cy5.5 antibody [CD8 Mouse Human PerCP-Cy5.5; Becton, Dickinson (BD), New Jersey, USA] for 30 min. For CD8+ T-cells, strict protocols for collection, storage, and sorting were followed to minimize contamination and ensure the highest quality of samples. These steps were carefully designed to maintain the integrity of the cells and ensure reproducibility across experiments. CD8-positive cytotoxic T-cells were sorted in 15 mL Falcon tubes (18 × 10^6^ cells were isolated on each Falcon tube) via the BD FACSAria™ III system. Thereafter, CD8+ cells were centrifuged at 1200× *g* for 10 min and then resuspended in fresh RPMI Medium [LM-R1638/500; Biosera, Cholet, France] containing 10% FBS, 1% pen-strep, 2% *v*/*v* Phytohemagglutinin [Phytohemagglutinin, Gibco, USA], and 10 ng/mL recombinant IL-2 [Animal-Free Recombinant Human IL-2; PeproTech, Cranbury, NJ, USA] for 72 h in 6-well plates. All experiments were performed under aseptic conditions. Lung cancer cell lines A549 and H1299, pre-incubated with 10 μΜ chloroquine [Sigma-Aldrich, USA] for 3 days, were seeded in 6-well plates in an approximate yield of 50 × 10^4^ cells/well (target cells). On the 3rd day, following removal of chloroquine, cancer cells were incubated with 20 μg/mL of an anti-PD-L1 monoclonal antibody [Durvalumab; AstraZeneca, Cambridge, UK] for one hour. Activated CD8+ T-cells were collected, centrifuged, and resuspended in fresh DMEM Low Glucose [Material: LM-D1102/500; Batch no: MS008W; ID no: MS008W100R; Biosera, Cholet, France] containing 20 μg/mL of an anti-PD-L1 monoclonal antibody. The T-cell culture was mixed with the cancer cell culture in a cancer/T-cell ratio of 1:3 and cultured for 72 h. Subsequently, cells were collected via trypsinization and washed twice with PBS 1× [Biosera, Cholet, France, Lot no: 017BS454]. For the evaluation of the apoptosis/necrosis of the cells, the Annexin V/PI [Catalog# 640914; Biolegend, San Diego, CA, USA] was utilized. Again, the analysis of the samples was conducted using the BD FACSAria™ III system and FlowJo V10 software was used for the analysis of all the data. The gating strategy was based on the unstained control condition of each cell line and all experiments were performed three times. The stop recording point was set at 30,000 events.

### 2.8. Immunohistochemistry

Tissues were collected after surgery using standardized methods, immediately preserved, and processed uniformly with time-matched protocols to avoid degradation, following the expertise of the Department of Pathology, to ensure optimal sample integrity and data processing. 

Double immunohistochemistry with antibodies recognizing the LC3A and HLA-class-I ABC molecules was performed in a series of 27 surgical specimens from patients with NSCLC. HLA-class-I (ABC) molecule expression was assessed with the mouse monoclonal antibody, ab70328 (abcam, Cambridge, UK), at a dilution of 1/200 and incubation of 60 min. The antibody was raised against the HLA-A extracellular domain and reacted with the heavy chain of human HLA-class-I A, B, and C (https://www.abcam.com/hla-class-1-abc-antibody-emr8-5-ab70328.html accessed on 23 September 2024). In a previous study of lung cancer [7], we showed that this provides a clear membrane staining in all normal lung tissue components (epithelial, stroma cells, vessels, and lymphocytes that act as an internal positive control for an immunohistochemistry assessment), while an extensive loss of membrane and cytoplasmic expression is evident in the majority of NSCLCs. Omission of the primary antibody is used as a negative control. LC3A was assessed with the rabbit polyclonal antibody (clone 1805a, Abgent, San Diego, CA, USA) raised against a synthetic peptide at the C-terminal cleavage site of the human cleaved MAP1LC3A. The double immunostaining technique was adapted from that previously reported by our group methods [10,11].

Briefly, immunohistochemistry was performed on formalin-fixed, paraffin-embedded (FFPE), 3 μm thick tissue sections. The sections were deparaffinized and placed in the EnVision FLEX (DAKO, Glostrup, Denmark) Target Retrieval Solution (pH 9.0), followed by microwaving (3 × 5 min.). Subsequently, sections were incubated with the primary rabbit polyclonal antibody to LC3A (AP1805a, Abgent, San Diego CA, USA) at a dilution of 1:100 overnight at 4 °C. The slides were washed with phosphate-buffered saline (PBS) and EnVision FLEX Peroxidase-Blocking Reagent was applied for 10 min in order to block endogenous peroxidase of the tissues. This was followed by washing with PBS and the EnVision FLEX HRP detection reagent was applied for 30 min. Following washing with PBS, EnVision FLEX DAB+ Chromogen was applied to the sections for 5 min. After two cycles of washing with PBS, the same procedure was followed for the immunostaining with the mouse monoclonal antibody for HLA-class-I (ab70328 clone: EMR8-5, abcam, Cambridge, UK) at a dilution of 1:200 for 1 h. At the end, the color was developed within 15 min of incubation with EnVision Flex HRP Magenta Substrate Chromogen System (DAKO, Glostrup, Denmark). The final step of the procedure involved washing with PBS and counterstaining with hematoxylin.

The assessment of the slides was performed by two independent experienced pathologists and discrepancies were resolved with the conference microscope. The percentage of cancer cells with positive HLA-class-I membranes, LC3A cytoplasmic, double HLA/LC3A expression, and finally, the percentage with a lack of any expression was recorded in all x200 optical fields. The mean score was used to characterize each tumor sample for each of the four parameters.

### 2.9. Statistical Analysis

Statistical analysis and graph presentation have been performed using the GraphPad Prism 8.0 version. Paired analysis was performed to compare groups of continuous variables and a *p*-value of <0.05 was considered for statistical significance.

## 3. Results

### 3.1. Exposure to Autophagy Blockers/Enhancers and IFNγ

**Figure 1:**
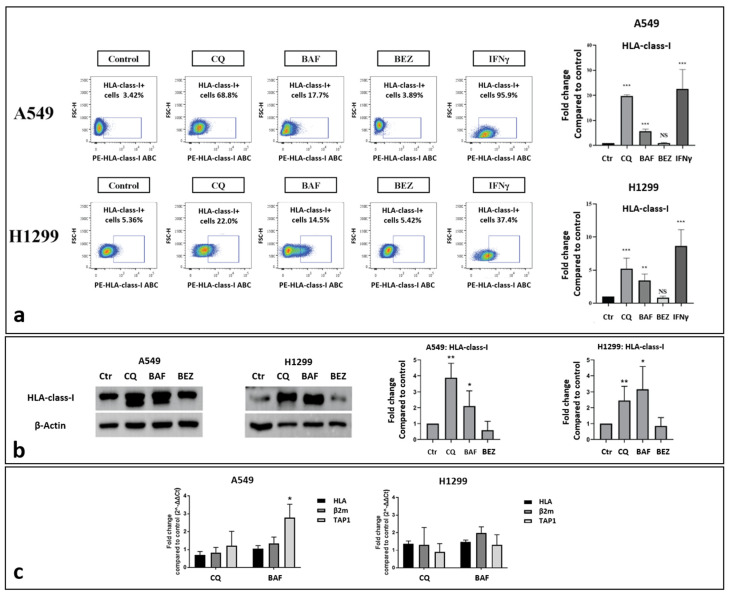
(**a**) Flow cytometry images and analysis of the expression levels of HLA-class-I protein on A549 and H1299 cell lines exposed for 72 h to 10 μΜ of chloroquine (CQ), 100 nM of bafilomycin A1 (BAF), 30 nM of NVP-BEZ235 (BEZ), and 25 ng/mL IFNγ (* *p* < 0.05, ** *p* < 0.01, *** *p* < 0.001). (**b**) Western blot images and analysis (fold increase compared to control ± SD) of HLA-class-I protein expression in A549 and H1299 lung cancer cell lines exposed to chloroquine (10 μΜ), bafilomycin (100 nM), and NVP-BEZ235 (BEZ) (30 nM) for 72 h. (**c**) Fold change compared to control (considered as 1) of HLA-class-I, β2-microglobulin (β2m), and TAP1 gene expression levels in A549 and H1299 lung cancer cell lines exposed to 10 μM chloroquine and 100 nM bafilomycin for 72 h.

Seventy-two-hour exposure of A549 and H1299 lung cancer cells to chloroquine (10 μM) and bafilomycin (100 nM) resulted in intense up-regulation of HLA-class-I molecule expression in the surface of the cell membranes. Flow cytometry analysis revealed that the percentage of A549 cancer cells with HLA-class-I expression in the chosen gate increased from 3.42% to 68.8% (19.75-fold increase; *p* < 0.001) and 17.7% (5.73-fold increase; *p* < 0.001) post-treatment with chloroquine and bafilomycin, respectively (Figure 1a). Similarly, HLA-class-I expression increased from 5.36% to 22.0% (5.22-fold increase; *p* < 0.001) and 14.5% (3.41-fold increase; *p* < 0.01) after incubation of H1299 cancer cells with chloroquine and bafilomycin, respectively (Figure 1a).

The inducing effect of IFNγ on HLA-class-I ABC expression levels was evident in both cell lines, as a strong increase was observed in the A549 (from 3.42% to 95.9%; 22.62-fold increase; *p* < 0.001) and H1299 (5.36% to 37.4%; 8.68-fold increase; *p* < 0.001) cell lines (Figure 1a). On the contrary, incubation with the autophagy enhancer NVP-BEZ235 did not induce any notable changes in either cell lines.

The Western blot analysis provided similar results, as shown in Figure 1b. The incubation of A549 cells with chloroquine and bafilomycin for 72 h increased the HLA-class-I protein band density by a mean (±standard deviation) of 3.51 ± 1.2, 2.11 ± 0.95 folds, respectively, see Figure 1b. The incubation of H1299 cells with chloroquine and bafilomycin enhanced the HLA-class-I protein expression by a mean (±standard deviation) of 2.31 ± 0.8 and 2.59 ± 0.7 folds, respectively, see Figure 1b. The incubation with NVP-BEZ235 did not have any effect on HLA-class-I expression in both lung cancer cell lines.

The RT-PCR analysis of the HLA-class-I, TAP1, and β2-microglobulin mRNA levels did not show any change in gene expression after exposure to chloroquine (10μΜ) or bafilomycin (100 nM) for 72 h (Figure 1c), although an increase in TAP1 mRNA levels was recorded in the A549 cell line after exposure to bafilomycin.

### 3.2. Time Dependence of Chloroquine Effect

The effect of chloroquine was time dependent, since six days of incubation resulted in a stronger increase in HLA-class-I levels compared to 3 days of incubation, from 3.70% to 60.5% (16.8-fold increase; *p* < 0.001) for the 3-day incubation and 75.7% (20.4-fold increase; *p* < 0.001) for the six days of incubation, and from 6% to 54.5% (8.57-fold increase; *p* < 0.001) for the three-day incubation and 63% (10.34-fold increase; *p* < 0.001) for the six-day incubation for A549 and H1299, respectively. Expression levels of HLA-class-I significantly decreased by two- to four-fold within three days after the removal of chloroquine from treated cancer cells (*p* < 0.001) (Supplementary Figure S1).

### 3.3. Effect of Other Novel Autophagy Inhibitors on HLA-Class-I+ Cells

The effect of various novel autophagy inhibitors on A549 and H1299 lung cancer cell lines was assessed after 72 h of exposure via flow cytometry. In the A549 cell line, incubation with SBI-0206965 (ULK-1 inhibitor) resulted in an increase in the percentage of HLA-class-I+ cells from 3.44% to 12.8% (3.49-fold increase; *p* < 0.001). Exposure to the PIKfyve inhibitor and the PROTAC PIKfyve degrader for 72 h resulted in a slight increase in the percentage of positive cells from 3.44% to 7.16% (1.89-fold increase; *p* < 0.05) and 7.88% (2.29-fold increase; *p* < 0.01), respectively. The same patterns were also observed in the H1299 cell line, where exposure to the ULK-1 inhibitor induced a strong increase in the percentage of positive cells from 5.47% to 27.5% (4.5-fold increase; *p* < 0.001), while exposure to the PIKfyve inhibitor and PROTAC PIKfyve degrader resulted in a slight increase in the positive cells to 11.2% (1.53-fold increase; *p* < 0.05) and 18.2% (3.23-fold increase; *p* < 0.01), respectively, see Supplementary Figure S2.

### 3.4. Effect on CD133+ Stem Cells

In order to examine the effect of chloroquine on the HLA expression of lung cancer stem cells, we conducted flow cytometry with double HLA-class-I and CD133 immunostaining. The CD133+ cell population composed 3.48% and 6.22% of the total A549 and H1299 cancer cell population, respectively. Chloroquine significantly enhanced the percentage of CD133+ cells in both cell lines to 22.4% (5.01-fold increase; *p* < 0.001) and 13.9% (2.38-fold increase; *p* < 0.01), respectively.

**Figure 2:**
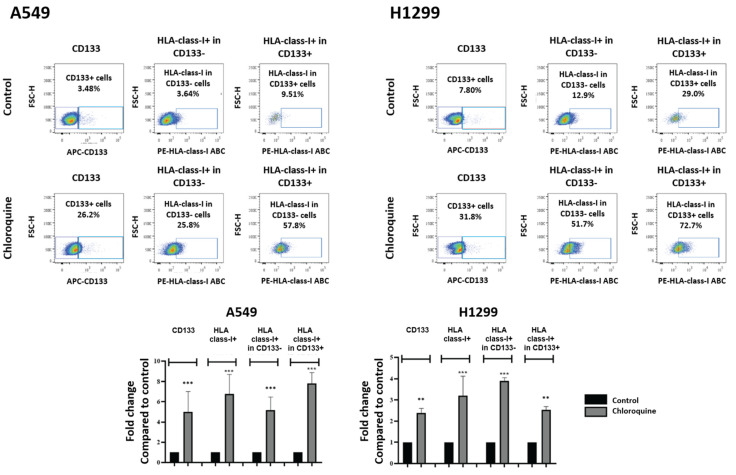
Flow cytometry images and analysis of CD133 and HLA-class-I-positive cells, and HLA-class-I-positive cells in either CD133-negative and -positive subpopulations of A549 and H1299 lung cancer cell lines, respectively, exposed to 10 μΜ of chloroquine (** *p* < 0.01, *** *p* < 0.001). Bars show the mean values and standard deviation. Percentages reported refer to the mean values.

In the A549 cell line, chloroquine incubation increased the percentage of HLA-class-I-expressing CD133- cells from a mean of 3.64% to 25.8% (7.82-fold increase; *p* < 0.001) and the percentage of HLA-class-I-expressing CD133+ cells from 9.51% to 57.8% (5.15-fold increase; *p* < 0.001) (Figure 2). In the H1299 cell line, chloroquine incubation increased the percentage of HLA-class-I-expressing CD133- cells from a mean of 12.9% to 51.7% (5.15-fold increase; *p* < 0.001), and the percentage of HLA-class-I-expressing CD133+ cells from 29.8% to 72.7% (2.53-fold increase; *p* < 0.01) (Figure 2).

### 3.5. Effect of MAP1LC3A Silencing

**Figure 3:**
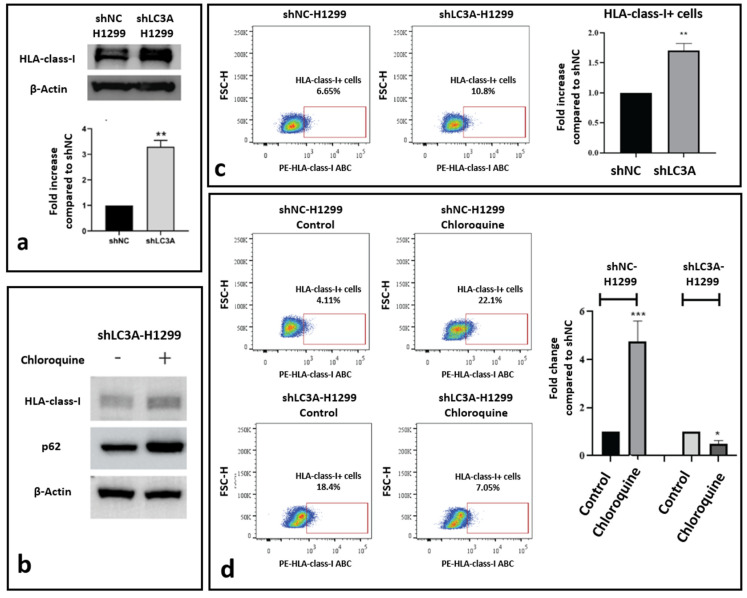
Effect of LC3A silencing on HLA-class-I expression in the H1299 lung cancer cell line. (**a**) Western blot image and densitometry of HLA-class-I protein band in H1299 cells transfected with non-coding sh (shNC) and shLC3A. (**b**) Western blot showing stable HLA-class-I protein levels in shLC3A-H1299 cells after exposure to 10 μΜ of chloroquine for 72 h. (**c**) Flow cytometry images and analysis of HLA-class-I-positive cells in shNC and shLC3A H1299 cell lines. (**d**) Flow cytometry images and analysis of HLA-class-I-expressing cells before and after exposure to 10 μΜ of chloroquine for 72 h (* *p* < 0.05, ** *p* < 0.01, *** *p* < 0.001). Bars show the mean values and standard deviation.

Comparing the expression levels of HLA-class-I protein in an H1299 cell line transfected with a non-coding plasmid vector (shNC-H1299) against the same cell line stably transfected with shLC3A-containing plasmid vector (shLC3A-H1299), a significant three-fold increase in the HLA-class-I protein was observed, as demonstrated by the Western blot analysis (Figure 3a). Upon exposure to chloroquine, the shLC3A-H1299 cells showed no change in HLA-class-I protein expression levels (Figure 3b). It is worth reporting that during post-treatment with chloroquine, we observed a two-fold increase in p62 protein levels, compatible with further autophagy blockage (Figure 3b).

Flow cytometry also revealed increased levels of HLA-class-I protein in shLC3A- H1299 lung cancer cells compared to the shNC-H1299. The percentage of HLA-class-I-positive cells was 6.65% and 10.8% (1.7-fold higher; *p* < 0.01), respectively (Figure 3c). Exposure to chloroquine resulted in a strong increase in the percentage of positive cells in the shNC-H1299 cell line (4.11% to 22%; 4.83-fold increase; *p* < 0.001) but had no stimulatory effect on the shLC3A-H1299 cells, where the levels decreased from 18.4% to 7.05% (2.06-fold decrease; *p* < 0.05), as shown in Figure 3d.

### 3.6. LC3A/HLA-Class-I Immunohistochemistry

**Figure 4:**
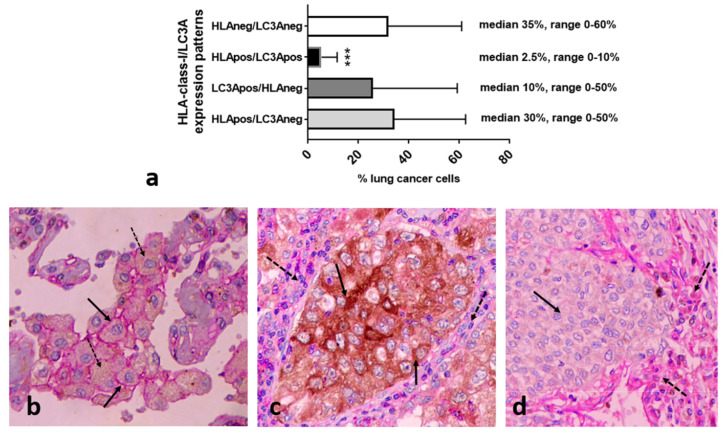
Analysis of the patterns of the percentage of LC3A/HLA-class-I expression in breast cancer tissue sections and typical double immunostaining images. (**a**) The percentage of breast cancer cells expressing the four different LC3A/HLA-class-I patterns (*** *p* < 0.001). (**b**) A simultaneous expression of LC3A/HLA-class-I (dotted and full arrows, respectively). (**c**) Single LC3A staining patterns in cancer cells (full arrow) in the context of stroma cells with HLA-class-I expression (dotted arrows). (**d**) The lack of LC3A and HLA-class-I expression by cancer cells (full arrows) in the context of stroma cells with HLA-class-I expression (dotted arrows).

The analysis of 27 non-small cell lung cancer cases, stained with double immunohistochemistry for LC3A and HLA-class-I, showed that the LC3A+/HLA+ cancer cell population was by far less conspicuous compared to the ones with the other three expression patterns (*p* < 0.01; Figure 4a). Figure 4b–d show a typical double immunostaining showing the simultaneous expression of LC3A/HLA-class-I, single staining patterns, and a lack of expression.

### 3.7. Lymphotoxicity, Chloroquine, and Anti-PD-L1

**Figure 5:**
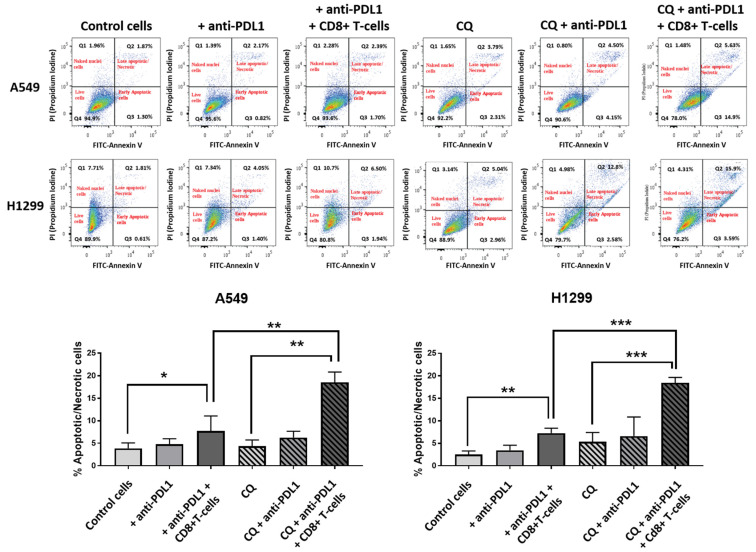
Analysis of apoptosis/necrosis in H1299 cells in control cells after incubation with anti-PD-L1 MoAbs, after pre-incubation with anti-PDL1 MoAbs followed by co-culture with activated CD8+ T-cells, after exposure to chloroquine, after incubation with chloroquine and anti-PDL1 MoAbs, and after pre-incubation with chloroquine and anti-PDL1 MoAbs followed by co-culture with activated CD8+ T-cells (* *p* < 0.05, ** *p* < 0.01, *** *p* < 0.001).

The effect of co-culturing lung cancer cells A549 and H1299 with activated cytotoxic CD8+ T-cells and an anti-PD-L1 monoclonal antibody, with or without pre-incubation with 10 μΜ chloroquine, was evaluated with flow cytometry as described in the methods. The percentage of early apoptotic and late apoptotic/necrotic cells was assessed (Figure 5).

In the A549 cell line, incubation with the anti-PD-L1 monoclonal antibody did not increase the mean % of apoptotic/necrotic cancer cells (3.8 vs. 4.8, *p* = 0.07), while the addition of activated CD8+ T-cells in the anti-PD-L1 pre-incubated cultures enhanced the mean % to 7.74% (*p* < 0.05), as shown in Figure 5. In experiments with pre-incubation with chloroquine, the anti-PD-L1 antibody produced a non-significant increase in the mean % of apoptotic/necrotic cells (4.35% vs. 6.29, *p* = 0.07), while the addition of CD8+ T-cells with the anti-PD-L1 antibody showed a strong increase in the mean value to 18.56% (*p* < 0.001), see Figure 5. The mean % was significantly higher in cells incubated with chloroquine, CD8+ T-cells, and anti-PD-L1 compared to cells incubated with only anti-PD-L1 and CD8+ T-cells (*p* < 0.01), see Figure 5.

Similar results were obtained in experiments with the H1299 cell line. Incubation with anti-PD-L1 did not result in a significant increase in the mean % of apoptotic/necrotic cells (2.5% vs. 3.4%, *p* = 0.2), while the addition of activated CD8+ T-cells in anti-PD-L1 pre-incubated cultures increased the mean % to 7.2% (*p* < 0.01), as seen in Figure 5. In experiments with cells pre-incubated with chloroquine, anti-PDL1 antibodies did not increase the mean % of apoptotic/necrotic cells (5.37% vs. 6.6%, *p* = 0.3). The addition of activated CD8+ T-cells strongly increased the percentage to 18.4% (*p* < 0.001), see Figure 5. The mean % of apoptotic/necrotic cells was significantly higher in cells incubated with chloroquine, CD8+ T-cells, and anti-PD-L1 compared to cells incubated with only anti-PD-L1 and CD8+ T-cells (*p* < 0.001), see Figure 5.

## 4. Discussion

T-cell-mediated cytotoxicity against cancer cells requires the presentation of non-self-antigens. Peptides produced by the proteasome-mediated degradation of abnormal proteins and oncoproteins can be recognized as targets by CD8+-activated T-cells once these are presented on the cancer cell surface through the HLA-class-I system [24]. Functional HLA-class-I proteins are formed in the endoplasmic reticulum following HLA heavy-chain assembling with the β2-microglobulin light chain and an antigenic peptide offered by the transporter associated with antigen-processing proteins (TAP1 and 2). Aminopeptidase (ERAP1 and 2) activity is also important to trim peptide length to 8-10 amino acids that are optimal for presentation [25]. Although all nucleated normal and cancer cells express HLA-class-I molecules, cancer cells may lose expression during the immuno-editing process, allowing them to evade immune surveillance. A variety of mechanisms are postulated to be involved, including HLA-related gene mutations and deletions, oncogene activation, and post-translational pathways [8]. In a previous study, we observed that about two-thirds of NSCLC tumors suffer from a total or extensive loss of membrane HLA-class-I expression and that microenvironmental conditions like hypoxia and acidity are involved in this process [7].

HLA-class-I repression may be a principal cause of immunotherapy failure [8]. In a study by Lee et al. in melanoma patients treated with ICIs, HLA-class-I down-regulation in biopsies was a common mechanism of resistance to PD-1 inhibitors [26]. Therapeutic policies that would effectively restore HLA-class-I expression would contribute to the enhancement of ICI efficacy. Recently, targeting autophagy has gained attention as a strategy to achieve this goal. In a study by Yamamoto et al., the degradation of MHC-class-I by autophagy was revealed as a central pathway of pancreatic cancer immune evasion [27]. In endometrial cancer, the autophagy protein LC3 interacts with and inhibits the function of MHC-class-I transactivator NLRC5, represses MHC expression, and promotes escape from antitumor immunity [28].

In the current study, we provide evidence that autophagy is a major target to enhance HLA-class-I expression in cancer cells and suggest certain autophagy-targeting agents, some of them already available in clinical practice for therapeutic repurposing and testing in cancer immunotherapy. The exposure of lung cancer cells to chloroquine, a blocker of auto-lysosomal fusion, and to bafilomycin, a blocker of lysosomal function, both drugs acting at a late step of the autophagy flux, resulted in strong induction of HLA-class-I expression. This expression was membranous, as suggested by flow cytometry results, which excludes an eventual non-functional accumulation in the cytoplasm. An additional finding was that autophagy blockage with chloroquine restored HLA-class-I expression in either differentiated or CD133+ stem cell populations. This finding is important, since stem cells, whether in normal or tumoral tissues, are postulated to evade immunity through the activation of various pathways, including HLA-class-I down-regulation [29,30]. CD133+ glioma cells have been shown to lack MHC-I expression that can be restored following incubation with IFNγ [31].

The silencing of the MAP1LC3A gene that encodes an autophagosome membrane protein necessary for cargo binding resulted in enhanced expression of HLA-class-I molecules. Double immunohistochemical staining of surgical NSCLC specimens showed that the cancer cell subpopulation expressing both LC3A and HLA-class-I protein was very limited in tumors, suggesting an inverse association between autophagy and HLA-class-I. Although chloroquine induced HLA-class-I expression in parental cells, this effect was not observed in shLC3A cancer cells, showing that the blockage of autophagy at an earlier step of autophagy formation may be sufficient to enhance HLA-class-I expression. This was further supported by experiments with a ULK1 inhibitor that blocks the first step of the recruitment of omegasomes in the endoplasmic reticulum, thus blocking autophagy at the initiation level. An interesting study by Deng et al. showed that ULK1 inhibition restores the suppression of antigen presentation, enhancing the T-cell infiltration of tumors and anti-PD1 immunotherapy activity [32]. Although autophagy blockage is expected to impede the degradation of abnormal neo-synthesized antigens, which may be a cause of the enhanced HLA-class-I expression after blocking the autophagic process at any step of its deployment [33], the suppression of LC3A per se may further enhance expression by hampering the internalization and degradation of membrane HLA molecules. Lapidated LC3 is necessary for the recruitment of AAK1 kinase and the internalization of HLA-class-I molecules [34]. Experiments with a blocker and a degrader of PIKfyve involved in endomembrane transport enhanced HLA-class-I expression. Thus, autophagy blockage may enhance HLA-class-I expression through multiple pathways involving both enhanced antigen availability and decreased membrane HLA degradation. Whether blocking autophagy may direct some proteins to the proteasome degradation pathway and increase immunopeptides is certainly a hypothesis that demands further investigation [35].

An additional mechanism would involve the transcriptional regulation of HLA-class-I-related proteins. Although such mechanisms are obscure as of yet, the discovery of the MHC-class-I transactivator CITA/NLRC5 and its direct inducibility by IFNγ has brought forward important aspects of the process [34]. Indeed, in the current study, ΙFNγ was a strong inducer of HLA-class-I expression. CITA/NLRC5 associates and activates the promoter of HLA-class-I, TAP1, and β2-microglobulin genes [36]. Although LC3 interaction with NLRC5 has been postulated to inhibit HLA-class-I gene expression and contribute to the immune escape of endometrial cancer cells [37], we did not find a significant association of autophagy blockage with chloroquine and the transcriptional activity of HLA-class-I, TAP1, and β2-microglobulin genes. A thorough investigation of links between autophagy, IFNγ, and CITA/NLRC5 is necessary to further elucidate the complex interactions between autophagy and antigen presentation in cancer [38].

The above-discussed experiments suggest that autophagy blockers may play an important role in antitumor activity through the restoration of HLA-class-I-mediated antigen presentation. To further explore this hypothesis, we conducted experiments with the incubation of lung cancer cells with activated CD8+ cells, confirming the expected increase in early/late apoptotic and necrotic cells. CD8+-mediated cytotoxicity was significantly enhanced in the presence of anti-PD-L1 MoAbs. The cytotoxic effect was further increased when cancer cells had been previously incubated with chloroquine, suggesting a therapeutic benefit of autophagy blockage-mediated HLA up-regulation in combination with ICIs. This synergy has been reported in mouse pancreatic cancer in vivo models, where chloroquine enhanced MHC-class-I expression and antitumor T-cell response under dual anti-PD-1/CTLA4 immunotherapy [28].

Limitations of the study include the verification of the HLA-inducing effect of autophagy blockers in real tumor microenvironmental conditions, often characterized by profound hypoxia and acidosis. Delicate in vitro experiments and in vivo treatment of animals with syngeneic experimental tumors or xenografts are demanded to further investigate the therapeutic value of autophagy blockers in combination with immune checkpoint inhibitors.

## 5. Conclusions

It is suggested that autophagy blockers acting either at late steps (e.g., chloroquine and bafilomycin) or at early steps (e.g., ULK1 inhibitors or LC3 silencing) of the autophagic process may have an important role in the restoration of HLA-class-I-mediated antigen presentation, eventually leading to the activation of dendritic cells, education of cytotoxic T-cells in lymph nodes, and ultimately enhancing antitumor cytotoxicity.

## Data Availability

All data, tissue material, stained slides, and analysis have been generated in the University Hospital of Alexandroupolis, are stored in the archives of our departments, and are available upon reasonable request.

## Supplementary Materials

The following supporting information can be downloaded at: https://www.mdpi.com/article/10.3390/cancers16193272/s1, Figure S1: HLA-class-I-positive A549 and H1299 cancer cells before and after incubation with chloroquine (10 μΜ) for three days, followed by an additional 3-day incubation without the drug, and finally, for six days with the drug. Figure S2: HLA-class-I-positive A549 and H1299 cancer cells before and after incubation with 2.5 μΜ of ULK-1 inhibitor, 5 μΜ of YM-201636 (PIKfyve inhibitor), and 300 nM of PIK5-12D (PROTAC PIKfyve degrader) for 72 h.
