# Supplementary material for: Autophagy Blockage Up-Regulates HLA-Class-I Molecule Expression in Lung Cancer and Enhances Anti-PD-L1 Immunotherapy Efficacy
Source: Cancers (Basel). 2024 Sep 26;16(19):3272. doi: 10.3390/cancers16193272 (PMC11476265; doi:10.3390/cancers16193272)
Supplement: Supplementary file 1 [file cancers-16-03272-s001.zip › cancers-3209620-supplementary.pdf]

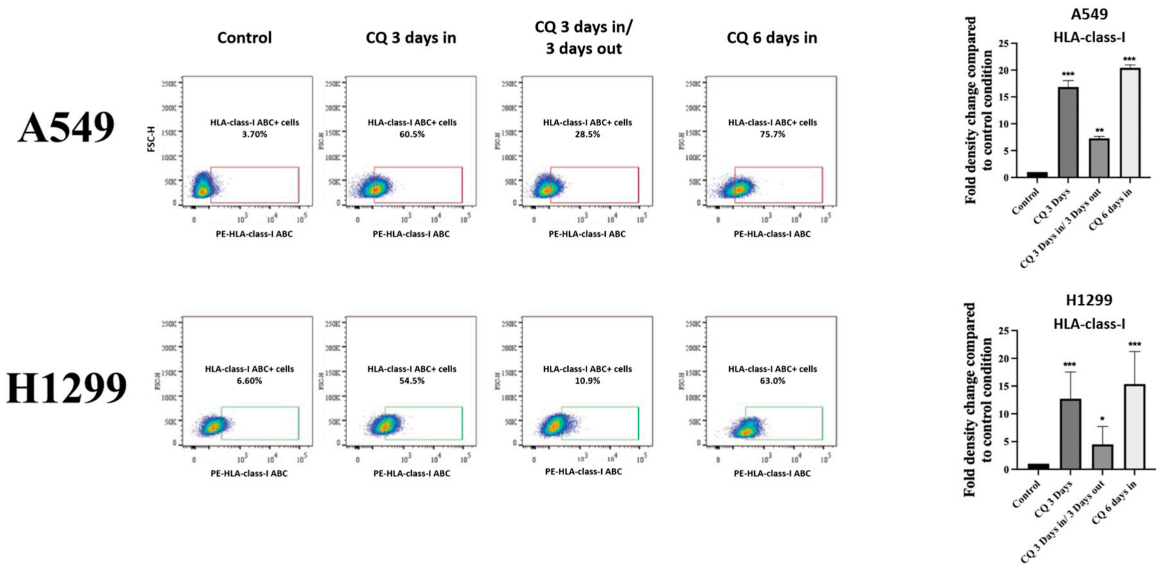

Supplemental Figure S1: HLA-class-I positive A549 and H1299 cancer cells before and after incubation with chloroquine (10 μM) for three days, for three days followed by an additional 3-day incubation without drug and, finally, for six days with the drug.

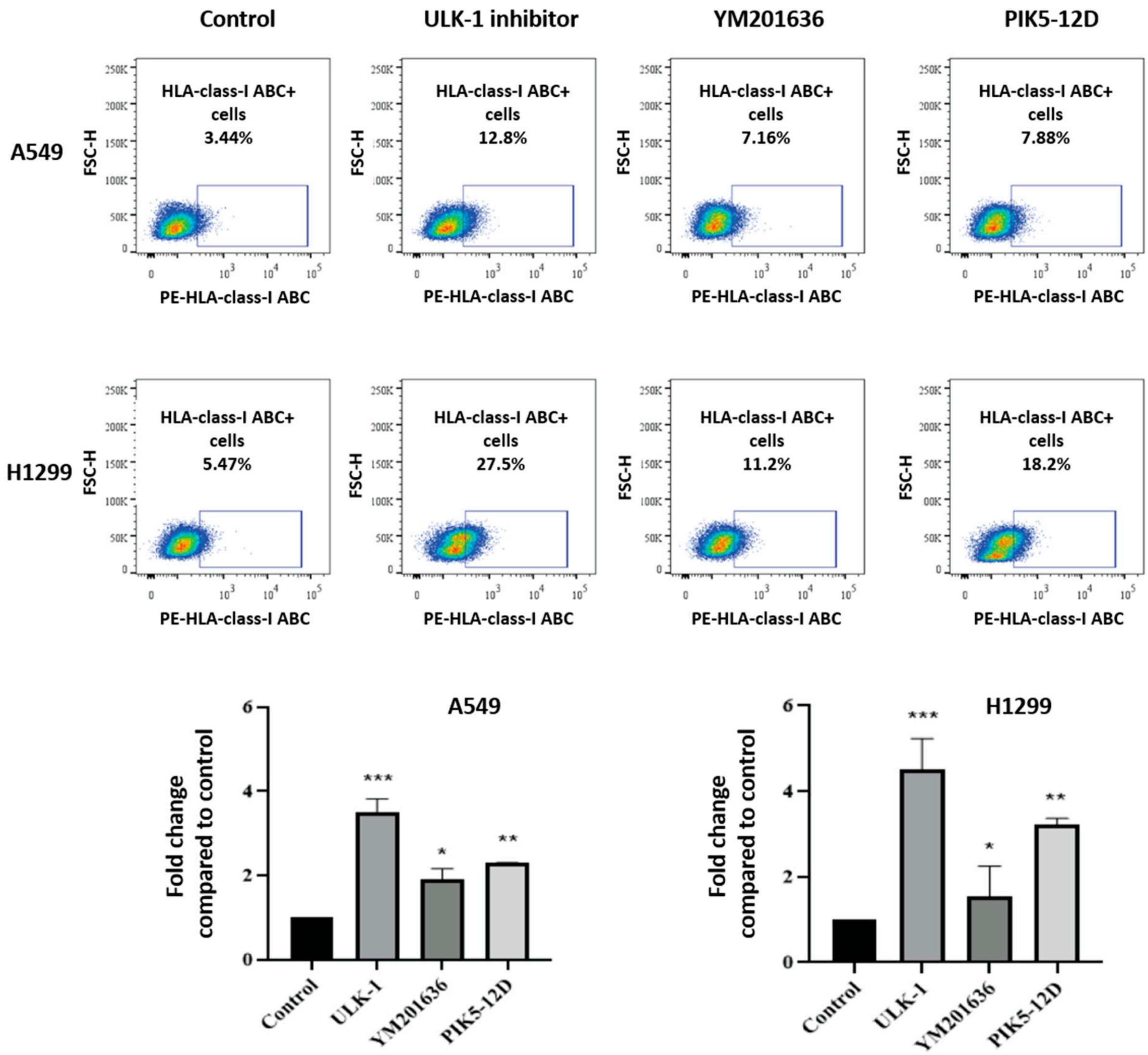

Supplemental Figure S2: HLA-class-I positive A549 and H1299 cancer cells before and after incubation with 2.5 µM of ULK-1 inhibitor, 5 µM of YM-201636 (PIKfyve inhibitor), and 300 nM of PIK5-12D (PROTAC PIKfyve degrader), for 72-hours.
